# A comprehensive study on machine learning models combining with oversampling for bronchopulmonary dysplasia-associated pulmonary hypertension in very preterm infants

**DOI:** 10.1186/s12931-024-02797-z

**Published:** 2024-05-08

**Authors:** Dan Wang, Shuwei Huang, Jingke Cao, Zhichun Feng, Qiannan Jiang, Wanxian Zhang, Jia Chen, Shelby Kutty, Changgen Liu, Wenyu Liao, Le Zhang, Guli Zhu, Wenhao Guo, Jie Yang, Lin Liu, Jingwei Yang, Qiuping Li

**Affiliations:** 1 Newborn Intensive Care Unit, Faculty of Pediatrics, the Seventh Medical Center of PLA General Hospital, Beiing, China; 2 The Second School of Clinical Medicine, Southern Medical University, Guangzhou, China; 3 School of Software, Tsinghua University, Beijing, China; 4 Department of Cardiology, Hunan Children’s Hospital, Changsha, China; 5 Department of Neonatology, Qingdao Women and Children’s Hospital, Qingdao, China; 6 Department of Neonatology, Tianjin Central Hospital of Gynecology Obstetrics, Tianjin, China; 7 Department of Neonatology, Guangdong Women and Children Hospital, Guangdong Neonatal ICU Medical Quality Control Center, Guangzhou, China; 8 Pediatric and Congenital Cardiology, Taussig Heart Center, Johns Hopkins School of Medicine, Baltimore, MD USA; 9 Department of Statistics and Data Science, BNU-HKBU United International College, Zhuhai, China; 10 Department of Neonatology, Nanfang Hospital, Southern Medical University, Guangzhou, China

**Keywords:** Bronchopulmonary dysplasia, Pulmonary hypertension, Machine learning, Prediction model, Oversampling

## Abstract

**Background:**

Bronchopulmonary dysplasia-associated pulmonary hypertension (BPD-PH) remains a devastating clinical complication seriously affecting the therapeutic outcome of preterm infants. Hence, early prevention and timely diagnosis prior to pathological change is the key to reducing morbidity and improving prognosis. Our primary objective is to utilize machine learning techniques to build predictive models that could accurately identify BPD infants at risk of developing PH.

**Methods:**

The data utilized in this study were collected from neonatology departments of four tertiary-level hospitals in China. To address the issue of imbalanced data, oversampling algorithms synthetic minority over-sampling technique (SMOTE) was applied to improve the model.

**Results:**

Seven hundred sixty one clinical records were collected in our study. Following data pre-processing and feature selection, 5 of the 46 features were used to build models, including duration of invasive respiratory support (day), the severity of BPD, ventilator-associated pneumonia, pulmonary hemorrhage, and early-onset PH. Four machine learning models were applied to predictive learning, and after comprehensive selection a model was ultimately selected. The model achieved 93.8% sensitivity, 85.0% accuracy, and 0.933 AUC. A score of the logistic regression formula greater than 0 was identified as a warning sign of BPD-PH.

**Conclusions:**

We comprehensively compared different machine learning models and ultimately obtained a good prognosis model which was sufficient to support pediatric clinicians to make early diagnosis and formulate a better treatment plan for pediatric patients with BPD-PH.

## Introduction

With the ongoing advancements in perinatal technology, there has been a remarkable increase in the success rate of treating very and extremely preterm infants diagnosed with bronchopulmonary dysplasia (BPD) [1]. However, some BPD infants may develop pulmonary hypertension (PH) which significantly impacts their mortality rate. Studies have demonstrated that the incidence of PH in preterm infants with BPD is high as 26.8% in other countries [2] with the mortality rate ranging from 14 to 38%, [3, 4] or even 50% in some countries [5, 6]. Even survivors still face short- and long-term adverse complications. The pathogenesis of BPD-PH remains unclear, and there is no safe and effective treatment for BPD-PH at present. Early prevention and timely diagnosis prior to pathological change are the key to reducing morbidity and improving prognosis [7]. How to identify infants at risk for BPD-PH has therefore become one of the hotspots of research for pediatric clinicians.

So far, there have been few studies reporting the establishment of BPD-PH predictive models [8]. Individual studies reporting the establishment of BPD-PH predictive models used regression coefficients to indicate the degree of correlation between risk factors and diseases, the algorithm that they used was relatively simple, and the results could not accurately give specific probability values. With the development of medical and health big data infrastructure, the form and quantity of medical data continue to improve, which gives us a chance to address the aforementioned technical caveats with a larger scale of training and more rigorous validation. In addition, to cope with the commonly seen unbalancing issue among real-world datasets, some machine learning techniques, such as oversampling could be tried out. Synthetic minority over-sampling technique (SMOTE) [9] is an oversampling method based on neural networks, which simulates the learning process of neural network to learn missing values and outliers. In this retrospective study, we intended to answer the following research questions by using the inpatient medical records from four different major tertiary-level medical centers in China:

- RS1 – Can machine learning methods support BPD-PH risk prediction in a more practical clinical setting across different organizations?
- RS2 – Can oversampling techniques such as SMOTE help improve the prediction results?

### Data collection

#### Patients

This study was approved by the research ethics board of the Seventh Medical Center of PLA General Hospital (No. 2022–02), and consisted with the Declaration of Helsinki. Informed consent was waived. The study population included very preterm infants (VPIs) with BPD who were born or admitted to the Seventh Medical Center of PLA General Hospital (Beijing, Chia), Qingdao Women and Children’s Hospital (Qingdao, China), Tianjin Central Hospital of Gynecology Obstetrics (Tianjin, China), and Guangdong Women and Children Hospital (Guangzhou, China) between August 1, 2015 and February 28, 2022. A total of 801 VPIs with BPD less than 32 weeks of gestational age were collected. Among these, there are 626 newborns in The Seventh Medical Center of PLA General Hospital, 69 newborns in Qingdao Women and Children’s Hospital, 59 newborns in Tianjin Central Hospital of Gynecology Obstetrics, and 47 newborns in Guangdong Women and Children Hospital. The following criteria were applied to construct the initial dataset:

Inclusion criteria were VPIs with gestational age less than 32 gestational weeks who were diagnosed with BPD. The diagnostic criteria and severity of BPD were based on the definition proposed in the conference of the National Institute of Child Health and Human Development in June 2000 [10], when their gestational age was corrected at 36 weeks.

Exclusion criteria were VPIs 1) at an admission age > 3 days or those who were not hospitalized beyond 36 weeks’ corrected gestation (due to discharge or death); 2) with congenital lung diseases, other anatomical abnormalities (such as diaphragmatic hernia or thoracic malformation), congenital heart disease except for patent ductus arteriosus (PDA), patent foramen ovale (PFO), atrial septal defect (ASD) and small interventricular septal defect (VSD) (defect diameter < 5 mm/m^2^ of body surface area) leading to PH.

VPIs with an admission age > 3 days or those hospitalized for < 36 weeks’ corrected gestation were considered valid subjects, knowing that admission age > 3 days may result in missing maternal and neonatal clinical data, while hospitalization < 36 weeks’ corrected gestation may affect the diagnosis of severity of BPD and PH. Therefore, a total of 40 cases of BPD infants were excluded and 761 cases of BPD infants were enrolled finally.

According to the results of Doppler echocardiography at least over 36 weeks’ corrected gestation, the patients were divided into BPD-PH group and BPD group.

### Clinical features

A total of 46 clinical features which are the same with variables were collected by reviewing the medical records during hospitalization, including maternal pregnancy factors, newborn clinical data, conditions of treatment and echocardiography-related information.

Maternal pregnancy factors included cesarean section, hypertension in pregnancy, gestational diabetes mellitus, intrauterine distress, preeclampsia, multiple births, natural conception, placental abnormality, placental abruption, premature rupture of the membrane (PROM), duration of PROM more than 18 h, and oligohydramnios.

Newborn clinical data included gestational age (week), birth weight (g), the severity of BPD, early-onset PH, 1-min Apgar score (A1), 5-min Apgar score (A5), meconium aspiration syndrome (MAS), amniotic fluid contamination, exclusive breastfeeding, neonatal respiratory distress syndrome (NRDS) more than II grades, ventilator-associated pneumonia [11], septicemia [12], necrotizing enterocolitis (NEC) requiring surgery, pulmonary hemorrhage [13], hemodynamically significant PDA [14], retinopathy of prematurity (ROP) more than II grades, small for gestational age (SGA), sex, age at initiated feeding (day), and time to achieve full enteral feeding (day).

Conditions of treatment included duration of invasive respiratory support (day), duration of nasal cannula oxygenation (day), duration of noninvasive respiratory support (day), corticosteroids used for treatment of BPD, usage of pulmonary surfactant (PS), a single dose, multiple doses, usage of vasoactive agents, usage of caffeine, and ligation of the PDA.

Echocardiography-related information included PFO, maximum diameter of PDA, VSD, ASD, the peak velocity of tricuspid regurgitant flow, velocity and direction of blood flow in PDA, shunt at the level of PFO, shunt at the level of VSD, and position of the ventricular septum.

These clinical features cover most of the factors that may affect the disease, allowing subsequent models to more accurately predict whether patients are at risk of developing the disease. The diagnostic criteria referred to related criteria [15, 16]. And all diagnostic and treatment times are before or at the time of correcting the gestational age of 36 weeks except BPD-PH. Early-onset PH was defined as PH occurring between 72 h and 14 days after birth [17]. BPD-PH was defined as PH occurring after 36 weeks' corrected gestation [18]. PH echocardiographic diagnostic criteria, ① Right ventricular systolic pressure (RVSP) > 35 mmHg (1 mmHg = 0.133 kPa); RVSP = (tricuspid valve flow velocity) ^2^ × 4 + right atrial pressure (usually 5 mmHg); ② RVSP/ sBP ratio > 0.5; ③ Any VSD or PDA with bidirectional or right-to-left shunt; ④ If there is no tricuspid valve regurgitation or shunt, then meet 2 of the following 3 criteria: ① Any degree of flattening of the ventricular septum; ②Right ventricular dilation; ③Right ventricular hypertrophy [19]. It is also noteworthy that the dataset exhibited an imbalance between the amount of BPD-PH data and the amount of data without PH.

## Methods

### Data preprocessing

The dataset was divided into two parts: 80% for training and 20% for validation. In the preprocessing stage of the study, several steps (including categorical feature encoding, missing value imputation and feature selection) were performed to prepare the training datasets for the machine learning methods. Firstly, the features of the BPD-PH status are described in **(**Table 1**)**. A *p*-value < 0.05 was considered statistically significant. 27 statistically significant indicators would be used in next steps. Secondly, categorical features were encoded into numerical form. The rules **(**Table 2**)** were utilized. Then, missing values in the datasets were addressed through imputation. The missing values in continuous features were replaced with the mean value of the respective feature, while the missing values in categorical features were replaced with the most frequent category. Lastly, in statistically significant indicators in single factor analysis, SelectKBest was utilized to select the most relevant features and evaluation function f_regression was applied to assess the correlation between the features and the target features [20]. The top 10 features with the highest scores **(**Table 3**)** are listed for analysis, constituting a better feature set for subsequent model training.

**Table 1:** Clinical characteristics between PH group and non-PH group

Feature	no-PH(*n* = 525)	BPD-PH(*n* = 83)	*p*-value
Newborn clinical data			
Gestational age (week)	28.2 ± 4.1	27.8 ± 3.6	< 0.001
Birth weight (g)	1260.0 ± 740.0	1050.0 ± 550.0	< 0.001
A1	5.5 ± 4.5	5.5 ± 4.5	0.069
A5	5.5 ± 4.5	5.5 ± 4.5	0.019
Age at initiated feeding (day)	13.5 ± 13.5	7.0 ± 7.0	0.22
The severity of BPD	2.0 ± 1.0	2.0 ± 1.0	< 0.001
Time to achieve full enteral feeding (day)	69.0 ± 69.0	75.5 ± 75.5	< 0.001
Early-onset PH	228(43.4%)	67(80.7%)	< 0.001
MAS	0(0.0%)	1(1.2%)	0.289
Amniotic fluid contamination	43(8.2%)	20(24.1%)	< 0.001
Exclusive breastfeeding	65(12.4%)	14(16.9%)	0.34
NRDS more than II grades	313(59.6%)	64(77.1%)	0.003
Ventilator-associated pneumonia	161(30.7%)	51(61.5%)	< 0.001
Septicemia	125(23.8%)	37(44.6%)	< 0.001
NEC requiring surgery	15(2.9%)	4(4.8%)	0.538
Pulmonary hemorrhage	46(8.8%)	25(30.1%)	< 0.001
Hemodynamically significant PDA	241(45.9%)	64(77.1%)	< 0.001
ROP more than II grades	62(11.8%)	21(25.3%)	0.002
SGA	12(2.3%)	7(8.4%)	0.008
Male	296(56.4%)	45(54.2%)	0.802
Maternal pregnancy factors			
Cesarean section	259(49.3%)	39(47.0%)	0.78
Hypertension in pregnancy	112(21.3%)	29(34.9%)	0.01
Gestational diabetes mellitus	137(26.1%)	22(26.5%)	1
Intrauterine distress	52(9.9%)	21(25.3%)	< 0.001
Preeclampsia	91(17.3%)	22(26.5%)	0.065
Multiple births	173(33.0%)	29(34.9%)	0.817
Natural conception	346(65.9%)	44(53.0%)	0.031
Placental abnormalities	77(14.7%)	24(28.9%)	0.002
Placental abruption	56(10.7%)	17(20.5%)	0.018
PROM	172(32.8%)	33(39.8%)	0.259
Duration of PROM more than 18h	134(25.5%)	29(34.9%)	0.096
Oligohydramnios	38(7.2%)	16(19.3%)	< 0.001
Conditions of treatment			
Duration of invasive respiratory support (day)	33.0 ± 33.0	91.5 ± 90.5	< 0.001
Duration of nasal cannula oxygenation (day)	45.5 ± 45.5	27.0 ± 27.0	0.093
Duration of noninvasive respiratory support (day)	52.0 ± 51.0	54.0 ± 54.0	0.001
Corticosteroids used for treatment of BPD	199(37.9%)	52(62.7%)	< 0.001
Usage of PS	487(92.8%)	80(96.4%)	0.323
A single dose	279(53.1%)	36(43.4%)	0.124
Multiple doses	208(39.6%)	44(53.0%)	0.029
Usage of vasoactive agents	274(52.2%)	66(79.5%)	< 0.001
Usage of caffeine	485(92.4%)	73(88.0%)	0.25
Ligation of the PDA	29(5.5%)	16(19.3%)	< 0.001
Echocardiography-related information			
Maximum diameter of PDA (mm)	2.9 ± 2.9	2.8 ± 2.8	< 0.001
PFO	508(96.8%)	82(98.8%)	0.505
VSD	22(4.2%)	8(9.6%)	0.063
ASD	96(18.3%)	23(27.7%)	0.063

The distribution of continuous features in the table is expressed by mean ± SD. The *p*-value based on t test for continuous features and chi-square for categorical features

*Abbreviations*: *PDA* patent ductus arteriosus, *BPD* bronchopulmonary dysplasia, *PH* pulmonary hypertension, *NRDS* neonatal respiratory distress syndrome, *NEC* neonatal necrotizing enterocolitis, *ROP* retinopathy of prematurity, *SGA* small for gestational age, *MAS* meconium aspiration syndrome, *PROM* premature rupture of the membrane, *PS* pulmonary surfactant, *VSD* ventricular septal defect, *ASD* atrial septal defect, *PFO* patent foramen ovale, *SD* standard deviation

**Table 2:** Value pairs before and after encoding

Before encoding	After encoding	Before encoding	After encoding
I	1	Mild	1
II	2	Moderate	2
III	3	Severe	3
IV	4	Yes	1
TRUE	1	No	0
FALSE	0

**Table 3:** Top 10 features with the highest regression scores

Feature	F_regression-Score	Rank
Duration of invasive respiratory support (day)	61.79	1
The severity of BPD	42.57	2
Early-onset PH	33.33	3
Pulmonary hemorrhage	31.34	4
Ventilator-associated pneumonia	29.16	5
Hemodynamically significant PDA	22.44	6
Usage of vasoactive agents	20.38	7
Ligation of the PDA	20.10	8
Amniotic fluid contamination	18.6	9
Corticosteroids used for treatment of BPD	61.79	10

*Abbreviations*: *BPD* bronchopulmonary dysplasia, *PH* pulmonary hypertension, *PDA* patent ductus arteriosus

### Oversampling

In our study population, the BPD-PH group accounted for 13% which was significantly lower than that of BPD group. Due to the lack of sufficient data, the classifier lacked the ability to characterize the BPD-PH group, making effective classification difficult. To reduce the impact of data imbalance and increase the accuracy of the model, oversampling of rare classes was used. For comparison, the number of samples in BPD-PH group was increased by using SMOTE.

### Classification model and evaluation indicator

In our study, multiple machine learning methods were used and compared. These methods included multivariate logistic regression [21, 22], decision tree [23], random forest [24] and neural network [22]. Their performance was evaluated and compared on the same dataset to determine which method performed best for prediction. To improve the training results, hyperparameters were adjusted separately for each method. The multivariate predictive model results were evaluated under the metrics accuracy, sensitivity, specificity and negative predictive value (NPV).

## Results

**Fig. 1:**
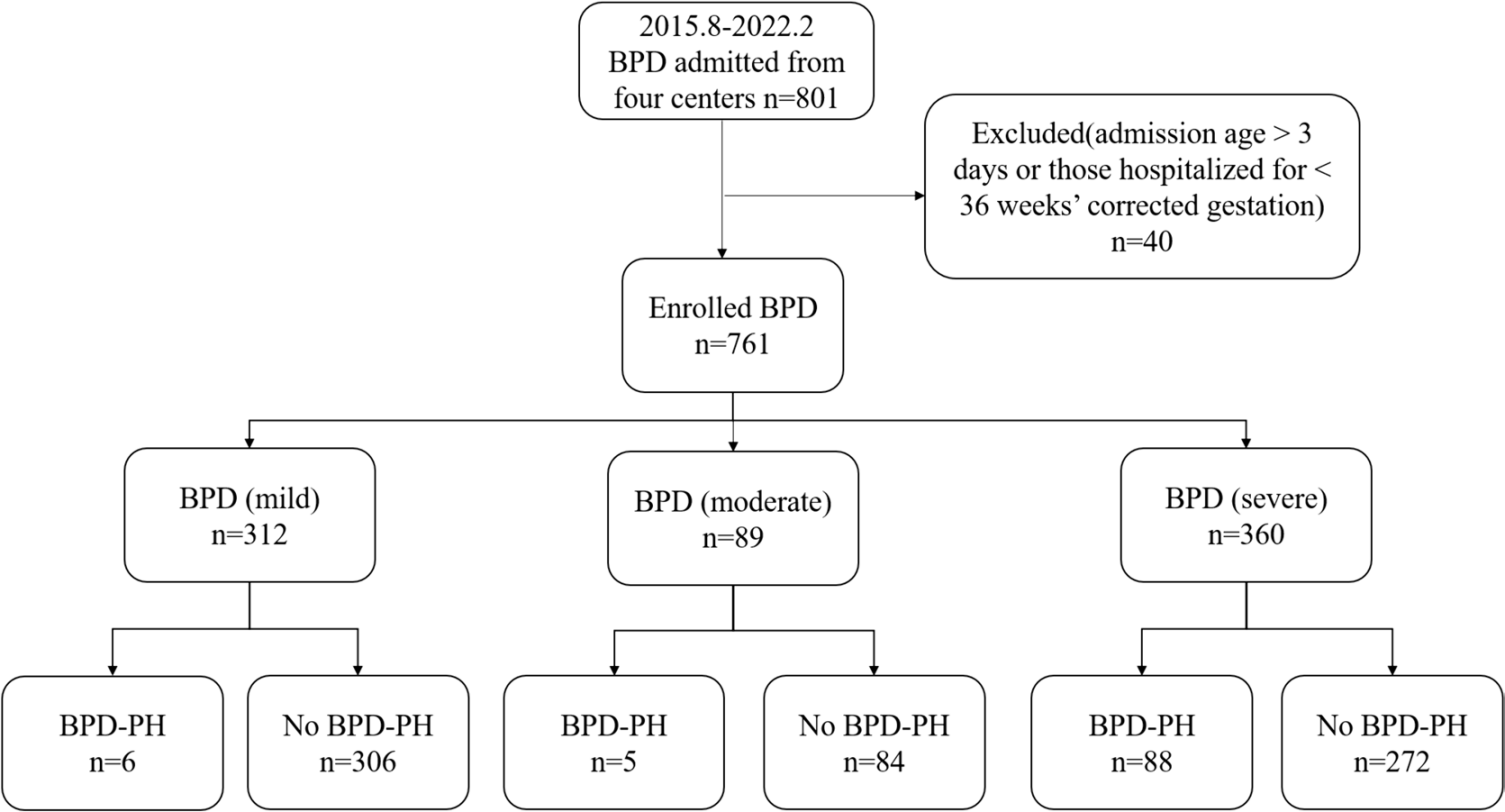
A composition chart of study participants

During the study period, 761 newborns (427 male and 334 female) with BPD were enrolled initially, among whom 99 newborns (13%) were later confirmed as having PH and 662 newborns (87%) as having no PH. The number of the 662 newborns with mild, moderate and severe BPD was 306, 84 and 272, respectively. The incidence of PH in grade mild to severe BPD newborns were 1.96% (6/306), 5.95% (5/84), and 32.35% (88/272), respectively (Fig. 1). The number of gestational weeks ranged from 24 to 32 weeks. The incidence of PH in 24–26, 26–28, 28–30, more than 30 gestational weeks at birth was 41.07% (23/56), 12.95% (44/365), 7.66% (20/261) and 15.19% (12/79), respectively. The number of birth weight ranged from 500 to 2000 g. The incidence of PH in 500–1000, 1000–1500,1500–2000 at birth weight was 17.30% (50/289), 10.76% (47/437) and 5.71% (2/35), respectively. The dataset was divided into two parts: 80% for training (608 cases) and 20% for validation (153 cases).

### Feature selection

Based on the scores and rankings (Table 3), 5 features were chosen for model training and functional evaluation including the duration of invasive respiratory support, the severity of BPD, ventilator-associated pneumonia, pulmonary hemorrhage, and early-onset PH.

### Training and evaluation

Training was performed by four machine learning methods and two sampling methods (original and SMOTE). Table 4 shows the results of different groups. When the model was trained directly using the training datasets without any data oversampling, it was possible to achieve a high accuracy and specificity. The accuracy scores, in logistics regression and neural network groups, could both achieve over 90%. However, due to imbalanced data, the classifier lacked the ability to characterize the BPD-PH group. So, the sensitivity, which indicates the detection rate of BPD-PH, tended to be low before oversampling. The accuracy scores in decision tree and random forest were lower than those in the previously mentioned groups, but the sensitivity was higher. The sensitivity of the model using SMOTE were significantly improved in logistics regression and neural network groups, but the effect was relatively small in random forest and decision tree groups.

**Table 4:** The evaluating indicators of the models

Sampling Method	Accuracy	Sensitivity	Specificity	NPV	AUC
**Logistic Regression**					
Original	0.922	0.438	0.978	0.937	0.928
SMOTE	0.85	0.938	0.839	0.991	0.933
**Decision Tree**					
Original	0.876	0.625	0.905	0.954	0.807
SMOTE	0.869	0.625	0.898	0.953	0.772
**Random Forset**					
Original	0.863	0.625	0.891	0.953	0.906
SMOTE	0.856	0.688	0.876	0.96	0.896
**Neural Network**					
Original	0.922	0.438	0.978	0.937	0.889
SMOTE	0.83	0.938	0.818	0.991	0.897

*Abbreviations*: *SMOTE* Synthetic minority over-sampling technique, *NPV* Negative predictive value, *AUC* Area under the ROC curve

Logistics regression and neural network groups exhibited the highest sensitivity (93.8%) and also relatively higher in other indicators. The final choice was the logistic regression model, because it provided interpretable results. The coefficients associated with each independent features could be easily interpreted as the log odds ratio, allowing us to understand the direction and magnitude of the effects. However, neural networks could capture complex patterns and relationships in large datasets. As the size of the data increased and the quality improved, neural networks may become more effective, so it is worthwhile to try out in our future work.

**Fig. 2:**
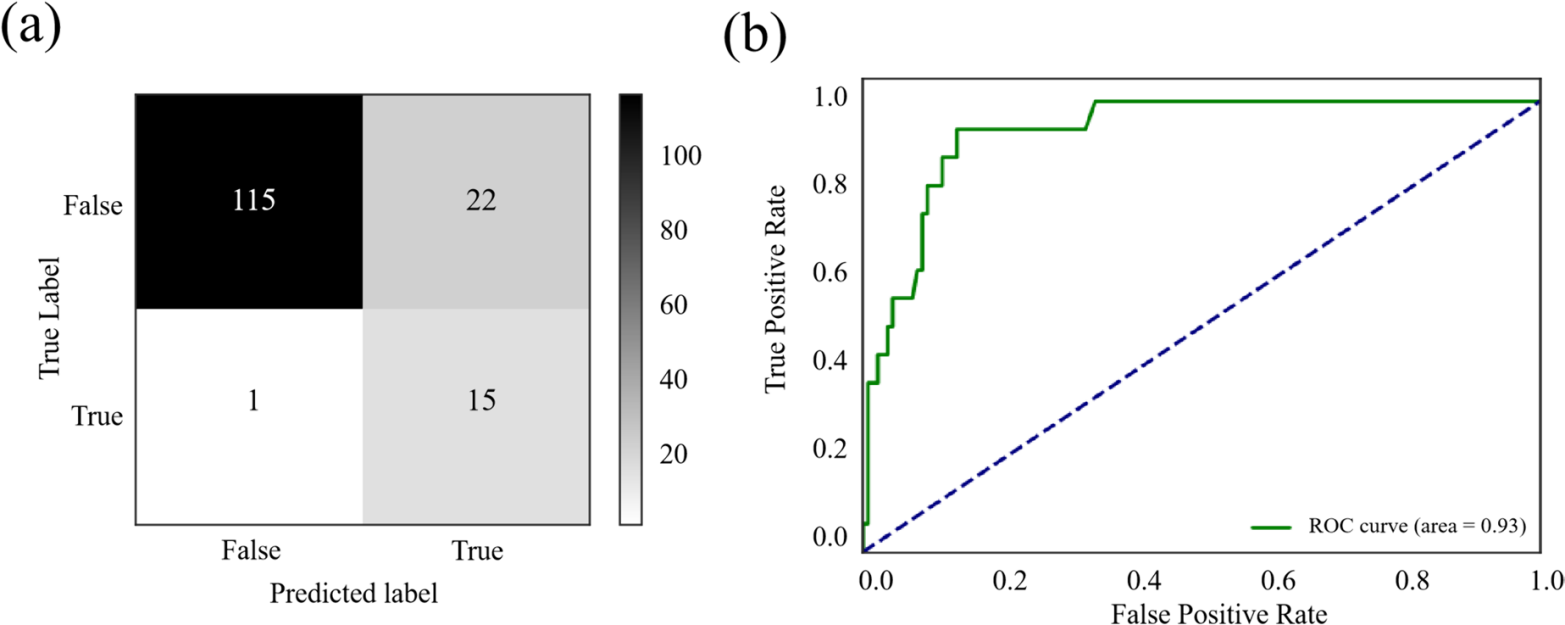
The performance of the selected model. (**a**) The confusion matrix and (**b**) the ROC curve. The AUC of this predictive model is 0.93

Figure 2 shows the result of the selection model. The area under the ROC curve (AUC) of this predictive model was 0.933. With the regression analysis, a predictive model was finally established.

$$\begin{array}{c}\text{logit}\left(\text{P}\right)=\text{ln}\left(\frac{\text{P}}{1-\text{P}}\right)=\\-4.850\\\begin{array}{c}+1.095\times\mathrm{The}\;\mathrm{severity}\;\mathrm{of}\;\mathrm{BPD}\;\left(\text{mild}=1,\;\mathrm{moderate}=2,\;\mathrm{severe}=3\right)\\+1.198\times\text{Early}-\mathrm{onset}\;\mathrm{PH}\;\left(\text{True}=1,\;\text{False}=0\right)\\+0.020\times\mathrm{Duration}\;\mathrm{of}\;\mathrm{invasive}\;\mathrm{respiratory}\;\mathrm{support}\;\left(\text{day}\right)\end{array}\\+0.703\times\text{Ventilator}-\mathrm{associated}\;\mathrm{pneumonia}\;\left(\text{True}=1,\;\text{False}=0\right)\\+0.948\times\mathrm{Pulmonary}\;\mathrm{hemorrhage}\;(\text{True}=1,\;\text{False}=0)\end{array}$$

**Fig. 3:**
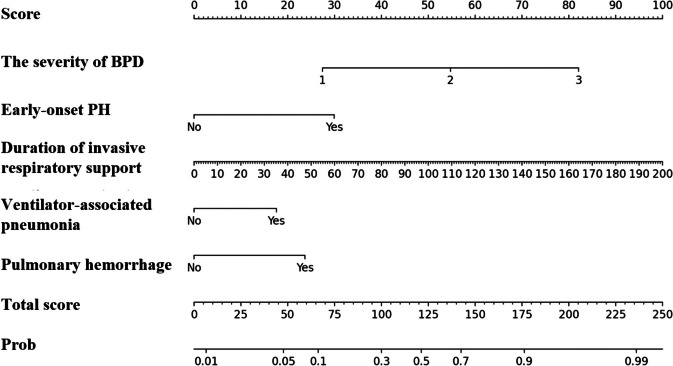
A nomogram predicting the risk of BPD-PH. To use, locate the corresponding value on each feature axis and draw a line straightup passing through the values to the 'Score' axis to determine the scores of features. Add the scores of each feature's values to obtain the 'Total score', and project it onto the 'Prob' axis to determine the probability of suffering from BPD-PH

If the value of logit(P) was greater than 0 (i.e. logit(P) = 0, odds = P/(1-P) = 1, P = 0.5), it was predicted as a positive class and otherwise as a negative class. A nomogram was developed based on the predictive model to improve the convenience of the model in clinical practice (Fig. 3).

## Discussion

In this study, we established a good model of predicting PH in VPIs with BPD by collecting the clinical data from four tertiary-level hospitals in China by using an artificial intelligence algorithm and different models to select the final indicators. By using advanced machine learning techniques, we could identify some important clinical factors associated with BPD-PH, such as, duration of invasive respiratory support (day), the severity of BPD, ventilator-associated pneumonia, pulmonary hemorrhage, and early-onset PH.

An ability to accurately predict BPD-PH and then taking measures to intervene were clinically important to avoid the bad survival outcome of infants with BPD. Presently, there exists a deficiency in a dependable tool that can accurately forecast BPD-PH during its initial stages. In this study, through using oversampling algorithms and multi-institutional clinical data, we finally had an overall good performance. These indicators are readily available clinically, which do not cause harm to children, and neonatologists can use the score corresponding to the formula to visually assess the risk of BPD-PH in VPIs. Scores greater than 0 should be considered a warning sign for developing BPD-PH. Compared with the study of Collaco et al. [25] our study had the same patient criteria for all data and did a calibration test. Compared with the study of Trittmann et al. [26] The AUC of our study was obviously higher and the data of our study were more accessible. In addition, we had a larger sample size and did verification. Although the AUC of our study was lower than that of the study by WANG et al. [27] our study was multi-institutional with a larger sample size. Multi-institutional study involves collecting data from different regions and healthcare institutions, thereby increasing the representativeness and generalizability of the samples. Additionally, multi-center data can help validate and verify the consistency and universality of research findings. Therefore, utilizing multi-center data for disease prediction provides more reliable and comprehensive information. The sample size was large enough in our study to obtain good results and we combined machine learning techniques. It is our hope that the model developed herein will help neonatologists to identify VPIs at risk of BPD-PH in time in the future and help pediatric clinicians to reduce the incidence and mortality of BPD-PH.

We found duration of invasive respiratory support (day) is clearly correlated with BPD-PH. Due to lung immaturity of VPIs, respiratory support is an important treatment for BPD patients. During the process of ventilator treatment, lung inflammation and capillary endothelial cell damage are more likely to occur in immature lung tissues. Vascular remodeling and pulmonary artery pressure increased [18]. Therefore, protective ventilation strategies should be adopted for preterm infants to minimize the injury caused by mechanical ventilation.

Ventilator-associated pneumonia, the severity of BPD, early-onset PH are also important factors in the occurrence of BPD-PH in our study. Ventilator-associated pneumonia is an independent risk factor for the development of BPD-PH, and is also a postnatal respiratory injury that disrupts the growth of pulmonary vessels and alveoli [28]. The presence of lung damage caused by prolonged ventilator use increases the number of inflammatory cells and mediators in the systemic circulation [29], and repeated inflammatory infections of the lungs make ventilator evacuation difficult, thus prolonging the duration of mechanical ventilation, which forms a vicious circle. Similarly, many studies have shown that moderate to severe BPD is associated with the development of BPD-PH [30–32], which is consistent with our study. Preterm infants with moderate to severe BPD often have severe lung developmental disorders, and long-term mechanical ventilation induced pulmonary inflammation and aggravates alveolar epithelial cell damage, resulting in PH finally. And a prospective study [17] also found that early-onset PH was significantly associated with BPD progression and BPD-PH in preterm infants. Therefore, early-onset PH is an independent risk factor for the development of BPD-PH [33], which is also supported by the model developed in this study. Animal studies have shown that an increase in pulmonary vascular pressure may directly damage vascular growth and alveolarization during lung development. In a study using a chronic intrauterine pulmonary hypertension model in fetal sheep, Grover et al. [34] found that chronic PH resulted in thickening of the pulmonary arteriole walls, decreased pulmonary arterial density, and simplified alveoli. These studies suggest that early-onset PH may play a role in the progression of BPD-PH by early impairing lung development and affecting vascular and alveolar development. These clinical factors are closely related and interact with each other, ultimately leading to the occurrence of BPD-PH.

In addition, our study suggests that pulmonary hemorrhage is also an important factor in the development of BPD-PH. This is an interesting finding, as few previous studies have reported pulmonary hemorrhage as a risk factor for BPD-PH. VPIs with pulmonary hemorrhage was mainly caused by other primary diseases such as infection and often had lower gestational age and body weight, and their lung development is absolutely immature. Previous studies [35] have shown that pulmonary hemorrhage children had a high incidence of BPD. So, these children often have higher requirements for ventilator parameters when pulmonary hemorrhage occurs, and the longer the usage of ventilator, the more severe the damage to the airway, pulmonary blood vessels and lung interstitium, and the more difficult to wean the ventilator, leading to an increased incidence of BPD-PH. Therefore, prevention and timely and effective treatment of pulmonary hemorrhage are beneficial in preventing the occurrence of BPD-PH.

This study has some strengths and weaknesses. In this study, we used the oversampling technique to address the issue of imbalanced positive and negative cases, finding that it could significantly improve the accuracy of the results. By using the oversampling technique, we artificially increased the number of minority class samples, ensuring a more balanced representation of both positive and negative cases in our dataset. SMOTE generated synthetic examples of PH-group, effectively increasing its presence in the training data. This helps the model learn and generalize better, improving its ability to accurately classify both positive and negative instances. Our study used four machine learning methods for training, and selected the optimal model through comparison. The advantage of using multiple machine learning methods lies in the ability to analyze and understand the data from different perspectives. Each method has its own underlying assumptions and algorithms, which can capture unique patterns and insights within the dataset. By comparing the results obtained from different methods, we can gain a comprehensive understanding of the data and identify the best-performing model. We found that decision tree and random forest performed relatively well before oversampling, but logistic regression performed better after oversampling. Both logistics regression and neural network performed well after oversampling and the final choice was the logistic regression model, because it provided interpretable results. However, as the size of the data increases and the quality improves, neural networks may become more effective, so it is worthwhile to try out in our future work. This study also had some limitations. Firstly, this model is mainly suitable for patients at or after the time of correcting the gestational age of 36 weeks, but for patients who are less than 36 weeks correcting the gestational age, we can also use it after inferring based on their condition. Then we can intervene with patients early or close follow-up should be conducted after these high-risk children patient is discharged. Secondly, our model involves four centers, but one center providing the bulk of the samples. This may limit the generalizability beyond the centers that provided the dataset. We need to continue to collect more positive cases from more centers for verification to improve the accuracy of the model and retry a neural network way to build a better predictive model. Thirdly, due to the unbalanced data, oversampling method was used in our study. However, oversampling may promote overfitting of the model to minority class samples, reducing generalizability beyond the dataset. We are going to carry out a prospective cohort study and follow-up to overcome this problem.

## Conclusion

In this study, we established a predictive model of BPD-PH by using five most significant of 46 clinical features. The predictive model could help clinicians to make early diagnosis and formulate better treatment plans for VPIs with BPD-PH in that it presented good performance for prediction and offered an AUC of 93.3%. Of course, larger-sample studies using other machine learning techniques to develop more BPD-PH predictive models are required to verify the findings and conclusions of the present study.

## Data Availability

The dataset analyzed during the current study is available from the corresponding author on reasonable request.

## Abbreviations

BPD: Bronchopulmonary dysplasia
PH: Pulmonary hypertension
SMOTE: Synthetic minority over-sampling technique
PDA: Patent ductus arteriosus
NRDS: Neonatal respiratory distress syndrome
NEC: Neonatal necrotizing enterocolitis
ROP: Retinopathy of prematurity
SGA: Small for gestational age
MAS: Meconium aspiration syndrome
PROM: Premature rupture of the membrane
PS: Pulmonary surfactant
VSD: Ventricular septal defect
ASD: Atrial septal defect
PFO: Patent foramen ovale
VPIs: Very preterm infants
NPV: Negative predictive value
AUC: Area under the ROC curve
SD: Standard deviation
